# A highly sensitive enzyme‑linked immunosorbent assay allows accurate measurements of brain‑derived neurotrophic factor levels in human saliva

**DOI:** 10.12688/f1000research.160304.2

**Published:** 2025-05-22

**Authors:** Fumie Akutsu, Shiro Sugino, Mitsuo Watanabe, Yves-Alain Barde, Masaaki Kojima

**Affiliations:** 1 FUJIFILM Wako Pure Chemical Corporation, Osaka, Japan; 2 School of Biosciences, Cardiff University, Cardiff, UK

**Keywords:** ELISA, BDNF, Saliva, Immunoassay, Analytical Method

## Abstract

**Background:**

Hitherto, BDNF levels in humans have been primarily measured in serum and/or plasma where these levels are readily measurable, but primarily reflect the content of BDNF in blood platelets. By contrast, previous attempts to measure BDNF levels in readily accessible human body fluids such as saliva have been complicated by a lack of sensitivity and/or specificity of BDNF ELISAs (see Discussion). Recently, the suitability of a highly sensitive BDNF ELISA assay was validated using mouse plasma and serum where conventional BDNF ELISA fail to detect BDNF. In this report, we demonstrate that BDNF levels in human saliva are extremely low, in the low pg/mL range, yet detectable in all saliva samples tested.

**Methods:**

Saliva samples were collected from healthy volunteers by a passive drool method. All samples were aliquoted and immediately frozen to keep at -80°C until use. At the time of use, the samples were thawed, centrifuged to remove any remaining particles and BDNF measurement conducted by using a previously validated BDNF ELISA assay (see below). Recombinant mature BDNF was used as a reference.

**Results:**

The intra-assay variability was in the range of CV = 1.8 to 4.9%. Saliva samples could be kept frozen at -80°C for 2 months until use for measurements, but more than 4 freeze and thaw cycles caused BDNF losses presumably due to structural change of the antigen. The measurements were not affected by the method of collection provided the samples were diluted at least 2-fold.

**Conclusions:**

The results indicate that human saliva samples collected in a non-invasive fashion can be used as a source of material to try and correlate BDNF levels with human conditions of interest. These results also confirm those of an independent study published recently using the same BDNF ELISA kit to measure BDNF levels in human saliva samples.

## Introduction

The role of Brain Derived Neurotrophic Factor (BDNF) in the function of the nervous system is well-established and a large body of work, primarily using mouse models, has long demonstrated this role to be essential for key aspects of brain function. ^1^ In humans, this essential role is supported by genetic studies including gene deletion and polymorphisms (for a recent review see Ref. 2). BDNF is also likely to be involved in a number of major neurological and psychiatric conditions including Major Depressive Disorder (MDD), ^3^ Alzheimer’s Disease, ^4^ Parkinson’s Disease, ^5^ and epilepsy. ^6^ What is less clear is the degree to which measurements of BDNF levels in accessible body fluids may be used to correlate these levels with brain function and dysfunction. While BDNF levels can be readily measured in human blood using traditional BDNF ELISAs, these levels primarily reflect the content of BDNF in blood platelets. ^7, 8^ Thus, whether or not these levels are informative with regard to brain function and dysfunction in humans remains uncertain. This important question has been difficult to answer conclusively because of a major difference between mice and humans with regard to the presence of BDNF in blood. In mice, the most widely used animal model to explore the function and dysfunction of the nervous system, megakaryocytes, the progenitor cells of platelets, do not express the *Bdnf* gene at significant levels, unlike in the case in humans. ^8^ Very recently, the minute levels of BDNF present in mouse blood could be quantified following the development of a much more sensitive BDNF ELISA. ^9^ These measurements could also be validated by incubating mouse serum sample with an anti-BDNF monoclonal antibody unrelated to the antibodies used in the BDNF ELISA. While this antibody markedly reduced the ELISA signal, such was not the case when similar experiments were performed with monoclonal antibodies specifically recognizing nerve growth factor (NGF) or neurotrophin-3 (NT3). Both NGF and NT3 are structurally related to BDNF and all 3 bind to the neurotrophin receptor p75 with similar affinities. ^10^ The BDNF levels determined in mouse blood were found to be about 3 orders of magnitude lower than those determined in human. Unlike is the case with human samples, no difference was found between BDNF levels in mouse plasma or serum, unlike is the case in humans. ^9^ This finding is consistent with the notion that BDNF in mouse blood does not originate from platelets, but from other sources, including the skeletal musculature as demonstrated by Fulgenzi et al. ^11^ Very recently, Ikenouchi et al. ^12^ conducted an independent study using the same BDNF ELISA described in the above and in an attempt to correlate BDNF levels in human saliva with psychological distress in healthcare workers ^12^ with the values in line with those reported here. Saliva samples can be readily collected and in a large number if needed, with minimum stress even for elderly patients. These straightforward points have attracted the attention of many in the biomedical community in the past as illustrated by a number of reports about measurement of BDNF in human saliva (see for example Zappella et al., ^13^ Biamonte et al., ^14^ Jasim et al., ^15^ and Zhang et al. ^16^). However, the BDNF values reported in these studies varied over a very wide range indicating that most BDNF measurement methods used thus far had either not the specificity and/or the sensitivity needed to reliably measure BDNF levels in human saliva, a conclusion already reached by Vrijen et al. ^17^ One recent exception was the use of a recently introduced and validated BDNF ELISA kit ^9^ that was independently used in a study conducted in parallel with ours (Ikenouchi et al., ^12^ see Discussion for details).

In the present study, we report the supporting data indicating that the highly sensitive BDNF ELISA assay can be used to accurately measure levels of BDNF in human saliva samples, including after sample storage.

## Methods

### Samples

Saliva samples were collected from 11 healthy volunteers at the company who did not have known significant health issues or drug usage. Written informed consents were obtained from all of the volunteers according to the company’s procedure which is set in accordance with Ethical Guidelines for Medical and Health Research Involving Human Subjects published by Ministry of Health, Labor, and Welfare, Japan. The participants were given a sterilized sample collection tube (2 mL cryovial (Salimetrics, Pennsylvania)) and instructed to rinse their mouth with a cup of water before saliva collection, saliva sample was collected into a sterilized sample collection tube by a passive drool method by using the Saliva Collection Aid (Salimetrics, Pennsylvania) according to the manufacturer’s instruction manual. All samples were collected in the same afternoon (noon to 5 pm) and then 6 to 10 mL of the samples were aliquoted into 1.5 mL Eppendorf Safe-Lock Tube (Eppendorf, Tokyo) and then immediately frozen and kept at -80°C until use. At a time of sample use, the samples were thawed at room temperature and then centrifuged at 1500 X g for 15 min to remove remaining particles (pre-treated samples).

Recombinant mature BDNF (PeproTech, Cranbury, New Jersey, USA) was purchased and used as a reference and standard material for the assays.

### Measurement of BDNF

BDNF measurement was conducted by using Mature BDNF ELISA kit Wako, High Sensitive (FUJIFILM Wako Pure Chemical Corporation, Osaka, Japan) according to the instructions provided with the kit. Briefly, samples were diluted 2-fold with the kit Buffer Solution. The BDNF standard stock solution was prepared by adding defined amount of purified water to the Mature BDNF Standard vial, and then a various concentration (0.000, 0.205, 0.512, 1.28, 3.20, 8.00, 20.0, 50.0, and 500 pg/mL) of standard solutions were prepared by diluting the 10 ng/mL Mature BDNF Standard stock solution with Buffer provided in the kit, according to the dilution scheme provided in the instruction. The solution initially filling the ELISA plate was discarded and the wells were washed 4 times with the Wash Solution (1X) included in the kit. The plate was inverted after each wash and gently tapped against clean paper towels to remove any excess liquid retained in the wells. 50 μL of diluted standard solution and of diluted samples were added to respective wells, with duplicate wells used for each standard and sample. After agitating the plate on a microplate mixer, the plate was then covered by Plate Seal (plastic film) and incubated for 2 hours at room temperature (20–25°C). After 2-hour incubation, the solution was discarded, and the wells were washed again 4 times with Wash Solution as above. 50 μL of Biotin-conjugated antibody solution was added to each well, the plate covered by Plate Seal and agitated and then incubated for one hour at room temperature. The solution was then discarded, and wells were washed 4 times with Wash Buffer as above. 50 μL of Peroxidase-conjugated Streptavidin Solution was added to each well, the plate was covered and incubated on for 30 min at room temperature. After a final series of 4 washes with the Wash Buffer, 50 μL of mixed luminescent reagents 1 and 2 (mixed at a ratio of 1:1 before use) were added to each well, the plate was placed on a shaker for 1 min and the luminescence was measured using a 96-well microplate reader Infinite200PRO MPlex from TECAN (Switzerland) at 10 min after the addition of the luminescent reagent. The standard solutions were used to generate a standard curve converting luminescence to BDNF concentrations used to determine the BDNF concentration in the experimental samples. All measurements were conducted twice (n=2) and average of 2 measurements was used for the evaluations.

## Results

### Spike test

Saliva samples were spiked with 0, 20, or 100 pg/mL of known concentrations of recombinant mBDNF (reference material) at a 9:1 ratio, and then measurements conducted according to the instruction provided in the ELISA kit. The yields of the spiked mBDNF ranged from 85.1 to 102.0%, within the manufacturer’s specifications (within ±15%). The distribution of BDNF levels in undiluted samples were 0.296 to 4.09 pg/mL (average = 1.04 pg/mL, SD = 1.09 pg/mL) ( Table 1).

**Table 1.** Recovery of spiked BDNF in saliva samples.

Saliva sample ID	Spiked BDNF (pg/mL)	Measured BDNF (pg/mL)	Recovered BDNF (pg/mL)	Yield (%)
#1	0	0.783	-	-
	20	18.4	17.7	88.5
	100	90.3	89.6	89.6
#2	0	0.327	-	-
	20	18.2	17.9	89.5
	100	91.6	91.3	91.3
#3	0	1.67	-	-
	20	22.1	20.4	102
	100	98.8	97.1	97.1
#4	0	0.512	-	-
	20	18.1	17.6	88.0
	100	86.9	86.4	86.4
#5	0	0.296	-	-
	20	18.7	18.4	92.0
	100	95.7	95.4	95.4
#6	0	0.393	-	-
	20	17.5	17.1	85.5
	100	85.5	85.1	85.1
#7	0	0.814	-	-
	20	18.4	17.6	88.0
	100	86.4	85.6	85.6
#8	0	4.09	-	-
	20	23.9	19.8	99.0
	100	91.1	87.0	87.0
#9	0	0.601	-	-
	20	18.6	18.0	90.0
	100	101	100	100
#10	0	0.852	-	-
	20	18.3	17.4	87.0
	100	86.3	85.4	85.4
#11	0	1.12	-	-
	20	18.5	17.4	87.0
	100	100	98.9	98.9

### Dilution linearity test

**Figure 1.**
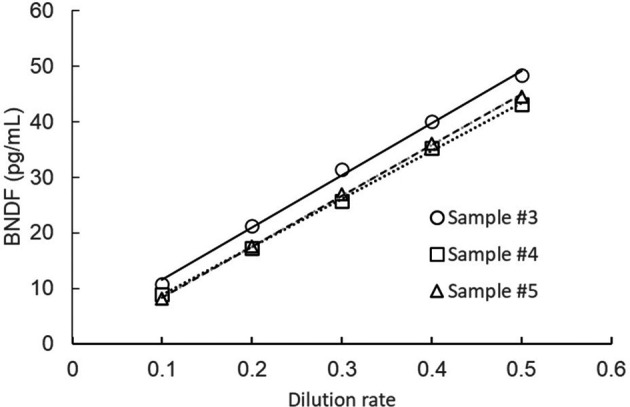
Dilution linearity test.

Samples were serially diluted using the buffer included in the kit, and BDNF levels measured. As shown in Figure 1, linearity was achieved when at least 2-fold sample dilution (one-to-one dilution) was conducted.

Samples were serially diluted by the buffer attached to the commercial kit.

### Precision

Samples were spiked with 4 or 40 pg/mL of recombinant mBDNF at a ratio of 9:1. The resulted samples were measured 10 times (n=10) using the ELISA kit to calculate the within precision of the assay. The results showed that intra-assay repeatability was calculated to be within a range of CV =1.8 to 4.9% as shown in Table 2.

**Table 2.** Assay precision of the mBDNF ELISA.

Sample ID	#2		#8		#9	
Spiked BDNF (pg/mL)	4.0	40.0	4.0	40.0	4.0	40.0
n	Measured BDNF (pg/mL)					
1	3.39	36.7	8.05	44.3	3.55	37.1
2	3.40	36.2	8.03	43.5	3.62	37.0
3	3.38	36.4	7.93	44.2	3.41	37.0
4	3.44	35.6	8.20	43.0	3.43	36.5
5	3.24	36.6	8.30	43.3	3.41	38.0
6	3.41	36.7	8.28	43.4	3.61	37.8
7	3.33	36.8	8.48	43.4	3.72	38.2
8	3.27	37.2	8.18	43.3	3.50	37.9
9	3.28	37.3	8.16	44.5	3.60	38.2
10	3.60	39.2	8.23	45.5	3.99	39.3
Mean	3.37	36.9	8.18	43.8	3.58	37.7
SD	0.10	0.95	0.16	0.77	0.18	0.82
CV (%)	3.1	2.6	1.9	1.8	4.9	2.2

### Sample stability

*Long-term stability*

Long term BDNF stability was assessed at 2 different temperatures, -20 and -80°C by using 6 saliva samples spiked with the recombinant mBDNF. They were kept at the designated temperatures for 2 or 3 months, then remaining BDNF levels were measured by the ELISA kit of this study.

As shown in Table 3, degradation of BDNF at -20°C was significant and 30-40% reduction was observed. By contrast, BDNF reduction during 2-month storage was minimal at -80°C and the changes were within 15% although one outlier showed 20% reduction, which was still within the boundary of the acceptable range of 80 to 120% shown in ICH HARMONISED TRIPARTITE GUIDELINE, “Validation of Analytical Procedures: Methodology: Text and Methodology. Q2(R1)” (2005). These data indicates that measurement should be performed within 2 months after saliva sample collection.

**Table 3.** Long term stability of BDNF in saliva.

a) Stored at -20°C						
ID	Day 0		2 months		3 months	
	BDNF (pg/mL)	(%)	BDNF (pg/mL)	(%)	BDNF (pg/mL)	(%)
Spiked sample A	1.86	100%	1.17	62.9%	0.46	24.6%
Spiked sample B	3.62	100%	2.23	61.6%	1.77	48.9%
Spiked sample C	5.76	100%	5.71	99.1%	4.05	70.3%
Spiked sample D	18.5	100%	14.2	76.8%	8.00	43.2%
Spiked sample E	29.6	100%	16.9	57.1%	11.3	38.2%
Spiked sample F	37.1	100%	24.0	64.7%	16.8	45.3%

b) Stored at -80°C						
ID	Day 0		2 months		3 months	
	BDNF (pg/mL)	(%)	BDNF (pg/mL)	(%)	BDNF (pg/mL)	(%)
Spiked sample A	1.86	100%	1.70	91.4%	0.88	47.5%
Spiked sample B	3.62	100%	3.43	94.8%	2.24	61.9%
Spiked sample C	5.76	100%	5.21	90.5%	5.05	87.7%
Spiked sample D	18.5	100%	16.4	88.6%	8.70	47.0%
Spiked sample E	29.6	100%	23.6	79.7%	16.0	54.1%
Spiked sample F	37.1	100%	34.7	93.5%	28.5	76.8%

### Repeated freeze and thawing test

**Figure 2.**
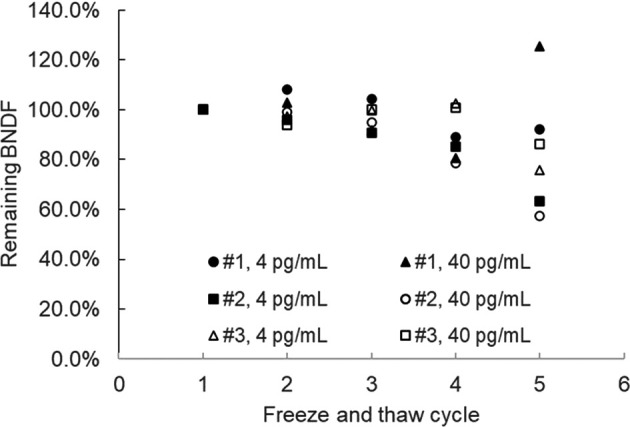
Effect of freeze and thaw cycles for the stability of BDNF in saliva samples.

Impact of repeated freeze and thawing was assessed by repeating freeze and thawing cycle of the spiked saliva samples for 5 time and the remaining BDNF levels were measured. As shown in Figure 2, more than 4 times freeze and thawing resulted in 15% or more BDNF reduction, therefore the maximum cycle number applicable to the frozen BDNF samples were determined to be 3 times or less.

### Evaluation of sample collection methods

In order to standardize the sample collection procedure, 2 types of saliva collection devices, Salivette made of cotton (Sartstedt, Nümbrecht, Germany) and SalivaBio Infant Swab (Salimetrics, PA, USA) were tested for the mBDNF recovery. The tested devices were soaked with the saliva samples (ID #2, 4, and #11) spiked with known concentrations of recombinant mBDNF and then samples were recovered by centrifugation (1000 X g, 2 min for Salivette and 1500 X g, 15 min for SalivaBio Infant Swab), according to the manufacturers’ instructions. The resulted samples were diluted 2-fold and then mBDNF levels were measured. The average recovery of the mBDNF with these commercially available saliva collection devices were 97.8 and 102.3% with the cotton (Salivette) and inactivated polymer (SalivaBio Infant Swab) devices, respectively ( Table 4).

**Table 4.** mBDNF recovery from swab samples.

Saliva sample ID	Spiked BDNF (pg/mL)	Measured BDNF (pg/mL)	Recovered BDNF (pg/mL)	Yield (%)
#2, cotton	0	0.224		
	20	18.6	18.4	92.0
	100	102	102	102
#2, inactivated polymer	0	0.309		
	20	20.5	20.2	101
	100	103	103	103
#4, cotton	0	0.432		
	20	18.2	17.8	89.0
	100	109	109	109
#4, inactivated polymer	0	0.580		
	20	23.0	22.4	112
	100	106	105	105
#11, cotton	0	0.804		
	20	18.0	17.2	86.0
	100	110	109	109
#11, inactivated polymer	0	0.980		
	20	20.9	19.9	99.5
	100	94.1	93.1	93.1

## Discussion

The main outcome of this study is that it confirms the notion that BDNF levels can be accurately quantified in human saliva samples with a commercially available, highly sensitive BDNF ELISA kit. The distribution of the BDNF levels in the saliva samples were 0.296 to 4.09 pg/mL, in line with the levels reported by Ikenouchi et al. ^12^ in a very recent study using the same BDNF ELISA. These levels are indeed extremely low and significantly below the detection limits of other commercially available ELISA kits, in agreement with conclusions reached by Vrijen et al. ^17^ These levels are also well below the limit of detection of notoriously insensitive methods such as Western blots. ^18, 19^ Also, previous attempts to increase the sensitivity of BDNF ELISA measurements by the use of for example BDNF polyclonal antibodies raise questions about the specificity of such assays. ^20, 21^ Not only do the levels of BDNF in human saliva reported in these previous studies vary greatly but also, they are 2 to 3 orders of magnitude higher than those reported here, i.e. in the sub-ng/mL to ng/mL range (see for example Mandel et al. ^20^, Bhat et al. ^21^ , or Tahiroglu et al. ^22^), and significant number (up to 35%) of saliva samples needed to be excluded from the analysis due to the lack of appropriate detection sensitivity in the case of Mandel et al. ^20^).

Obviously, saliva samples can be readily and repeatedly collected for BDNF level determinations and possible correlations explored with various neurological and psychiatric conditions. While the origin of BDNF in saliva is unclear the study by Ikenouchi et al. ^12^ demonstrated that there is no correlation between the levels of mBDNF in saliva and plasma. One possible source of BDNF in saliva samples are the sensory nerves innervating the buccal cavity including the tongue and gingiva as the levels of BDNF in sensory afferents are comparatively high (for review, see 2). Given that it is now possible to measure BDNF levels in human saliva, it would be interesting to know whether BDNF levels in saliva correlate with a range of physiological and pathological conditions of interest using appropriate study designs.

## Conclusion

The results of this study using a highly sensitive BDNF ELISA demonstrate that saliva samples can be used to measure BDNF levels accurately in readily accessible human samples like saliva, thus confirming the very recent results of Ikenouchi et al. ^12^ Correlations can now be readily explored between BDNF levels in human saliva and a range of physiological and pathological conditions of interest, including exercise, ageing, depression and neurodegeneration.

## Ethical considerations

Collection of the saliva samples from the volunteers was approved at the Expedited 27th Fujifilm Wako Pure Chemicals Life Science Ethics Review Committee with the approval number of #LS 011 on February 3, 2022. The company’s Ethics Review Committee was set in accordance with Ethical Guidelines for Medical and Health Research Involving Human Subjects published by Ministry of Health, Labor, and Welfare, Japan, and written informed consents were obtained from all of the volunteers.

## Roles

**Fumie Akutsu**: Data curation; Formal analysis; Investigation; Validation; Writing—original draft; Writing—review & editing. **Shiro Sugino**: Conceptualization; Investigation; Writing—review & editing. **Mitsuo Watanabe**: Writing—review & editing. **Yves-Alain Barde**: Writing—review & editing. **Masaaki Kojima**: Conceptualization; Funding acquisition; Investigation; Supervision; Validation; Writing—review & editing.

## Data Availability

Dataset for the tables and figures (Saliva validation data for manuscript final.xlsx) is available at https://www.doi.org/10.6084/m9.figshare.27978699.v1. ^23^ This project contains the following underlying data:
1. Dataset for the tables and figures (Saliva validation data for manuscript_final.xlsx) Data are available under the terms of the Creative Commons Attribution 4.0 International license (CC-BY 4.0).
