# Supplementary material for: A highly sensitive enzyme‑linked immunosorbent assay allows accurate measurements of brain‑derived neurotrophic factor levels in human saliva
Source: F1000Res. 2025 May 22;14:161. Originally published 2025 Feb 4. [Version 2] doi: 10.12688/f1000research.160304.2 (PMC12206289; doi:10.12688/f1000research.160304.2)
Supplement: Peer review history

# Reviewer report: Reviewer response for version 2

**DOI:** 10.5256/f1000research.181316.r388831

**Published:** 2025-07-22

**Authors:** Emad Al-Dujaili

**Affiliations:** 1 Queens Research Medical Institute, University of Edinburgh, Edinburgh, Honorary Professor, UK

The assay seems to be very well conducted and followed the guidelines to develop and evaluate an immunoassay method. I have the following the comments

1. The researcher does not have to freeze and thaw 4 times. He must prepare aliquots and use one at a time!
2. Add some important results to the abstract such as the specificity and sensitivity.
3. Collection and handling of saliva samples were properly conducted; however, the number of participants was small.
4. What is the lower detection limit and sensitivity of this ELISA?
5. Is there some specificity data performed? I did not see any.
6. In the abstract, the authors said that saliva samples were collected by a passive drool method. However, in the results, they use the Salivette made of cotton (Sartstedt, Nümbrecht, Germany) and SalivaBio Infant Swab (Salimetrics, PA, USA). These methods are different! Can the authors explain?
7. The discussion is short and require further clarifications.  The nature of the volunteers, and samples from males verses females etc. What are the applications of this assay?

Is the work clearly and accurately presented and does it cite the current literature?

Yes

If applicable, is the statistical analysis and its interpretation appropriate?

Not applicable

Are all the source data underlying the results available to ensure full reproducibility?

Yes

Is the study design appropriate and is the work technically sound?

Yes

Are the conclusions drawn adequately supported by the results?

Yes

Are sufficient details of methods and analysis provided to allow replication by others?

Partly

Reviewer Expertise:

BiochemistryNutrition and DieteticsNutritional Biochemistry, EndocrinologyPharmacy

I confirm that I have read this submission and believe that I have an appropriate level of expertise to confirm that it is of an acceptable scientific standard, however I have significant reservations, as outlined above.

# Reviewer report: Reviewer response for version 2

**DOI:** 10.5256/f1000research.181316.r390268

**Published:** 2025-06-28

**Authors:** Sedat Yildiz

**Affiliations:** 1 University of Inonu, Malatya, Turkey

I thank to the authors for this simple yet important presentation. As BDNF is associated with many physiological/pathological functions in the body, its non-invasive determination in saliva samples provides a valuable tool for reasearch and developments in associated areas. 

 The authors have used FUJIFILM Wako's chemiluminescence assay. Although features of this assay is given in the website of the producer, some details need to be given in this manuscript. These are:

 1. Please provide reference curve that you have obtained and used for calculation of the results. The readership would like to see the shape of that curve. My imagination, after reading the data in the commercial website, is that the curve is very linear and suitable for determination of low levels of BDNF (such as in saliva). 

 2. Please provide cross-reactivity of the antibody/test towards the similar molecules such as NGF-beta and NT, as presented in the commercial website. This will help readership to understand specificity of the antibody/test.

 3. Please provide the data about the specific recognition of BDNF by the  test as revealed by the BDNF knockout mice. This further provides data about reliability of the test developed. 

 4. Please state (in materials methods section, under mmeasurement of BDNF) thar this test is a chemiluminescence test. So that, the readership can understand it is a sensitive test kit.

 5. If it is feasable to you, please provide data that increased saliva level does not result in an interference in the assay. Although dilution study is quite parallel, we need to know matrix effect as the standards are prepared in buffer instead of saliva or artificial saliva. In order to solve this issue, please add increasing amounts of saliva to reference curve and check whether the linearity and parallelism with original standard curve continues. If it is not feasible for you, you can omit this comment.

 There are some othe minor corrections that needed to be carried out:

 1. In the abstract section, please remove "see discussion" part as it is not necessary in the abstarct

 2. In the abstract section, beginf the results with the mean levels of the BDNF, rather than the validation data. Please provide the levels first, then the validation. Because, as a reader, I  would like to see the real/quantitative results in the saliva, as it is said to be very low compared to the blood samples. 

 3.In the results section, please add the reference curve first, as explained above.

 4. In the discussion section, please use a a careful language when referring to the products of the other companies. In that respect, in terms of research oriented kits, no one should expect similar levels accross the products of the different companies. Because, this generally is due to the purity and structure of the test types. But this does not mean that the other test kits are not able to measure the analyte. The level might be different but the measurement might be more accurate. As far as I understand from the producer's web site, this kit is not a "diagnostically approved kit". Therefore, "different levels accross the companies" should not be the backbone of the discussion section. Rather, the usability of these test kits might be discussed or the areas where all these test kits might be used should be explained more.

 4.In the first sentence of the discussion, rather than using "confirm", please use "provides evidence" as there should be much more comprehensive studies that the data presented in the current study.

 5. In the discussion section, please remove the term "notoriously" as it is not an academic explanation. Please use more direct language like "... detection  by the methods like Western blots".

 6. Please use a careful language for the test kits that use polyclonal antibodies. Because, in ELISA, sometimes the best results are obtained by the use of polyclonal antibodies or cocktail of the monoclonal antibodies. 

 7. In conclusions section, please remove "...thus confirming ...". There is no need for the last part of this sentence. There should not be a "confirming" situation taking into account the variables in the test systems and biology itself.

 8. Please remove the words "readily" from the conclusion section. Instead, the terms "easily" or "non-invasively" should be preferred.

Is the work clearly and accurately presented and does it cite the current literature?

Yes

If applicable, is the statistical analysis and its interpretation appropriate?

Yes

Are all the source data underlying the results available to ensure full reproducibility?

Yes

Is the study design appropriate and is the work technically sound?

Yes

Are the conclusions drawn adequately supported by the results?

Yes

Are sufficient details of methods and analysis provided to allow replication by others?

Yes

Reviewer Expertise:

Immunoassay development, stress physiology, antibody production, assay validation, antibody or antigen tagging with enzymes, immunoaffinity purification,

I confirm that I have read this submission and believe that I have an appropriate level of expertise to confirm that it is of an acceptable scientific standard.

# Reviewer report: Reviewer response for version 2

**DOI:** 10.5256/f1000research.181316.r386656

**Published:** 2025-06-09

**Authors:** Bijorn Omar Balzamino

**Affiliations:** 1 IRCCS Fondazione Bietti, Rome, Italy

The author claims that other kits show very variable values ​​of bdnf in saliva, according to him, very high and therefore influenced by various factors, including the non-specificity of the kit used.

 I appreciate the author's response, but I still can't understand how he can justify that this product is more specific than an R&D mab248 duo-set for example. Any kit used for research has a certificate of specificity of the marker, specifying no cross-reactivity with other similar markers. if the author then wants to make this kit usable in diagnostics, the results must be demonstrated differently.

Is the work clearly and accurately presented and does it cite the current literature?

Partly

If applicable, is the statistical analysis and its interpretation appropriate?

Yes

Are all the source data underlying the results available to ensure full reproducibility?

Partly

Is the study design appropriate and is the work technically sound?

Yes

Are the conclusions drawn adequately supported by the results?

Partly

Are sufficient details of methods and analysis provided to allow replication by others?

Yes

Reviewer Expertise:

Research and development laboratory for biochemical, molecular and cellular applications in ophthalmological sciences

I confirm that I have read this submission and believe that I have an appropriate level of expertise to state that I do not consider it to be of an acceptable scientific standard, for reasons outlined above.

# Reviewer report: Reviewer response for version 2

**DOI:** 10.5256/f1000research.181316.r386655

**Published:** 2025-06-06

**Authors:** Elizabeth A Thomas

**Affiliations:** 1 University of California Irvine, Irvine, CA, USA

I have no further comments.

Is the work clearly and accurately presented and does it cite the current literature?

Partly

If applicable, is the statistical analysis and its interpretation appropriate?

Yes

Are all the source data underlying the results available to ensure full reproducibility?

Yes

Is the study design appropriate and is the work technically sound?

Yes

Are the conclusions drawn adequately supported by the results?

Yes

Are sufficient details of methods and analysis provided to allow replication by others?

Yes

Reviewer Expertise:

biomarkers, peripheral biofluids (plasma, saliva), neurodegenerative diseases

I confirm that I have read this submission and believe that I have an appropriate level of expertise to confirm that it is of an acceptable scientific standard.

# Reviewer report: Reviewer response for version 1

**DOI:** 10.5256/f1000research.176178.r371741

**Published:** 2025-04-04

**Authors:** Elizabeth A Thomas

**Affiliations:** 1 University of California Irvine, Irvine, CA, USA

The paper “A highly sensitive enzyme‑linked immunosorbent assay allows accurate measurements of brain‑derived neurotrophic factor levels in human saliva” presents important assay details for quantifying BDNF levels in saliva samples. These include spike and recovery data for the saliva matrix, the effects of different saliva collection protocols, linearity of dilution in the saliva matrix, precision and reproducibility of the BDNF measures and information regarding sample stability under long-term storage and the effects of multiple freeze thaws on BDNF detection. These are all essential considerations to show that saliva can be used for BDNF measurements. Saliva is a non-invasive biofluid that has gained recent attention for use in a wide range of human studies; hence knowing assay specifics for quantification of a very popular physiological peptide by ELISA has important consequences for others wanting to measure BDNF in saliva.

 The authors list previous studies that have quantified BDNF in human saliva samples and state that “BDNF values reported in these studies varied over a very wide range, indicating that most BDNF measurement methods used thus far had either not the specificity and/or the sensitivity needed to reliably measure BDNF levels in human saliva”.  Aside from using different ELISA assays, it is also very likely that these past studies used different saliva collection methods and storage conditions, which the authors show can affect BDNF levels in saliva. Hence, the current findings could possibly explain some of the variability observed in past studies. This is another relevant aspect of the work.

 A few other corrections/suggestions:

 -In the Introduction, they include Nakagawa et al. (ref 12) and Kikuchi et al., (ref 13) as human saliva studies, when these were carried out in rats. These should be omitted, but there are several other studies that have measured BDNF in saliva samples in humans that could be mentioned. 

 -In particular, one past study (Mandel A et a., 2011) also had worked out optimized conditions for another BDNF ELISA (although not as in depth as the current paper) and this study should be mentioned somewhere.

 -In the Abstract, it is not necessary to mention the mouse study: “Recently, the suitability of a highly sensitive BDNF ELISA assay was validated using mouse plasma and serum where conventional BDNF ELISA fail to detect BDNF”. The fact that it was validated in mouse is not as important as the fact that the assay works well in human saliva.

 -The introductory text regarding the origin of plasma/serum-derived BDNF levels is interesting, as the author make the suggestion that BDNF levels measures in blood might not inform about brain dysfunction in humans. Two previous studies (Ikenouchi  A et al., 2023 and Gutierrez A et al., 2020). shown that saliva and plasma levels of BDNF are not correlated, possibly suggesting that saliva measures could be more relevant. This is worth mentioning in the Discussion.

 -The time frame (i.e. 2 pm to 5 pm) of saliva sample collection should be stated.  Also, the amount of saliva (i.e. 1 ml) collected should be stated.

Is the work clearly and accurately presented and does it cite the current literature?

Partly

If applicable, is the statistical analysis and its interpretation appropriate?

Yes

Are all the source data underlying the results available to ensure full reproducibility?

Yes

Is the study design appropriate and is the work technically sound?

Yes

Are the conclusions drawn adequately supported by the results?

Yes

Are sufficient details of methods and analysis provided to allow replication by others?

Yes

Reviewer Expertise:

biomarkers, peripheral biofluids (plasma, saliva), neurodegenerative diseases

I confirm that I have read this submission and believe that I have an appropriate level of expertise to confirm that it is of an acceptable scientific standard, however I have significant reservations, as outlined above.

# Response

**Published:** 2025-04-29

**Authors:** Fumie Akutsu

**Affiliations:** FUJIFILM Wako Pure Chemical Corporation, Japan

Dear Dr. Thomas,

 Please find our responses written below.

**Reviewer’s comments:**

 The paper “A highly sensitive enzyme‑linked immunosorbent assay allows accurate measurements of brain‑derived neurotrophic factor levels in human saliva” presents important assay details for quantifying BDNF levels in saliva samples. These include spike and recovery data for the saliva matrix, the effects of different saliva collection protocols, linearity of dilution in the saliva matrix, precision and reproducibility of the BDNF measures and information regarding sample stability under long-term storage and the effects of multiple freeze thaws on BDNF detection. These are all essential considerations to show that saliva can be used for BDNF measurements. Saliva is a non-invasive biofluid that has gained recent attention for use in a wide range of human studies; hence knowing assay specifics for quantification of a very popular physiological peptide by ELISA has important consequences for others wanting to measure BDNF in saliva.

 The authors list previous studies that have quantified BDNF in human saliva samples and state that “BDNF values reported in these studies varied over a very wide range, indicating that most BDNF measurement methods used thus far had either not the specificity and/or the sensitivity needed to reliably measure BDNF levels in human saliva”.  Aside from using different ELISA assays, it is also very likely that these past studies used different saliva collection methods and storage conditions, which the authors show can affect BDNF levels in saliva. Hence, the current findings could possibly explain some of the variability observed in past studies. This is another relevant aspect of the work.

 A few other corrections/suggestions:

 -In the Introduction, they include Nakagawa et al. (ref 12) and Kikuchi et al., (ref 13) as human saliva studies, when these were carried out in rats. These should be omitted, but there are several other studies that have measured BDNF in saliva samples in humans that could be mentioned.

 1. We agree to your comment to remove Nakagawa et al (ref 12) and Kikuchi et al (ref 13), since their studies are not for human but for rat saliva BDNF levels.

 We included the Tahiroglu et al. in the Discussion section (new ref 22) as the most recent report discussing measurement of salivary BDNF.

 -In particular, one past study (Mandel A et al., 2011) also had worked out optimized conditions for another BDNF ELISA (although not as in depth as the current paper) and this study should be mentioned somewhere.

 2. We add some description about Mandel A et al in Discussion: “and significant number (up to 35%) of saliva samples needed to be excluded from the analysis due to the lack of appropriate detection sensitivity in the case of Mandel et al ^20^.”

 -In the Abstract, it is not necessary to mention the mouse study: “Recently, the suitability of a highly sensitive BDNF ELISA assay was validated using mouse plasma and serum where conventional BDNF ELISA fail to detect BDNF”. The fact that it was validated in mouse is not as important as the fact that the assay works well in human saliva.

 3. Although the reviewer suggests removing the sentence about the mouse study in the Abstract, we believe this is an important improvement in measurement of BDNF in various samples which was not done by commercially available BDNF assays in the past. So, we would like to keep the sentence in the Abstract as it specifically refers to validation, an all-important aspect. Sensitivity is of course key but so is validation. Readers may be interested to go back to the study we cite in our manuscript and check for themselves what is meant by validation. Briefly, beyond the reduction of the BDNF signal using a BDNF monoclonal antibody not used in the BDNF ELISA. the finding that BDNF levels in mouse plasma and serum are indistinguishable is key. Indeed, the process of platelet degranulation leads to the release of a number of cytokines and growth factors from platelets and the finding that the BDNF values remain unchanged compared with the plasma values is an important aspect of the validation.

 -The introductory text regarding the origin of plasma/serum-derived BDNF levels is interesting, as the author make the suggestion that BDNF levels measures in blood might not inform about brain dysfunction in humans. Two previous studies (Ikenouchi A et al., 2023 and Gutierrez A et al., 2020). shown that saliva and plasma levels of BDNF are not correlated, possibly suggesting that saliva measures could be more relevant. This is worth mentioning in the Discussion.

 4. Per reviewer’s suggestion, we modify the discussion section: “Obviously, saliva samples can be readily and repeatedly collected for BDNF level determinations and possible correlations explored with various neurological and psychiatric conditions. While the origin of BDNF in saliva is unclear, the study by Ikenouchi et al. ^12^ demonstrated that there is no correlation between the levels of mBDNF in saliva and plasma. One possible source of BDNF in saliva samples are the sensory nerves innervating the buccal cavity including the tongue and gingiva as the levels of BDNF in sensory afferents are comparatively high (for review, see 2). Given that it is now possible to measure BDNF levels in human saliva, it would be interesting to know whether BDNF levels in saliva correlate with a range of physiological and pathological conditions of interest using appropriate study designs.”  

 -The time frame (i.e. 2 pm to 5 pm) of saliva sample collection should be stated.  Also, the amount of saliva (i.e. 1 ml) collected should be stated.

 Is the work clearly and accurately presented and does it cite the current literature?

 5. We modify the part about the sample collection by adding the time frame and amount of the saliva sample collection to the original manuscript: All samples were collected in the same afternoon (noon to 5 pm) and then 6 to 10 mL of the samples were aliquoted into 1.5 mL

# Reviewer report: Reviewer response for version 1

**DOI:** 10.5256/f1000research.176178.r367703

**Published:** 2025-02-24

**Authors:** Bijorn Omar Balzamino

**Affiliations:** 1 IRCCS Fondazione Bietti, Rome, Italy

**Report**

 1 **. Importance of the idea**.

- In this short paper the authors analyze the effectiveness of a new ELISA kit for BDNF, using saliva as sample.
- Unfortunately, the idea is not new and there are several kits on the market for the analysis of BDNF from, which have also been used for the study of BDNF in saliva (see Nakagawa et al., 2019; Ye et al., 2020, Jasim et al., 2020; Kikuchi et al., 2020; Zappella et al., 2021; Biamonte et al., 2022 and Zhang et al., 2024) for example.
- The method section is well written so for statistical results.

2. **Recommending a Revision and Resubmission:**

- I think that the entire work should be set up differently, specifying better how other kits already exist on the market and how the BDNF has already been evaluated with these kits, therefore it’s more likely to describe a comparison between them.
- The results and discussion are well written and justified, the only thing that is not well illustrated is the abstract part where it is said that BDNF is not detectable in saliva, and this, as previously stated, is not true.

Is the work clearly and accurately presented and does it cite the current literature?

Partly

If applicable, is the statistical analysis and its interpretation appropriate?

Yes

Are all the source data underlying the results available to ensure full reproducibility?

Partly

Is the study design appropriate and is the work technically sound?

Yes

Are the conclusions drawn adequately supported by the results?

Partly

Are sufficient details of methods and analysis provided to allow replication by others?

Yes

Reviewer Expertise:

NA

I confirm that I have read this submission and believe that I have an appropriate level of expertise to state that I do not consider it to be of an acceptable scientific standard, for reasons outlined above.

## References

[1] : Effect of social isolation stress on saliva BDNF in rat. *J Oral Sci* .2019;61(4) : 10.2334/josnusd.18-0409 516-520 10.2334/josnusd.18-0409 31631095

[2] : Inflammatory biomarkers and growth factors in saliva and gingival crevicular fluid of e-cigarette users, cigarette smokers, and dual smokers: A pilot study. *J Periodontol* .2020;91(10) : 10.1002/JPER.19-0457 1274-1283 10.1002/JPER.19-0457 32052441 PMC9014419

[3] : Daytime changes of salivary biomarkers involved in pain. *J Oral Rehabil* .2020;47(7) : 10.1111/joor.12977 843-850 10.1111/joor.12977 32277715

[4] : Hypertriglyceridemia-induced brain-derived neurotrophic factor in rat submandibular glands. *J Oral Biosci* .2020;62(4) : 10.1016/j.job.2020.08.003 327-335 10.1016/j.job.2020.08.003 32931901

[5] : Relaxation Response in Stressed Volunteers: Psychometric Tests and Neurotrophin Changes in Biological Fluids. *Front Psychiatry* .2021;12: 10.3389/fpsyt.2021.655453 655453 10.3389/fpsyt.2021.655453 34220571 PMC8247444

[6] : Circulating and Salivary NGF and BDNF Levels in SARS-CoV-2 Infection: Potential Predictor Biomarkers of COVID-19 Disease-Preliminary Data. *J Pers Med* .2022;12(11) : 10.3390/jpm12111877 10.3390/jpm12111877 36579579 PMC9697501

# Response

**Published:** 2025-02-26

**Authors:** Fumie Akutsu

**Affiliations:** FUJIFILM Wako Pure Chemical Corporation, Japan

Dear reviewer,

*“We never claimed that measuring BDNF levels in human saliva is a new idea. Our intention was instead to demonstrate that this is a feasible objective using a BDNF ELISA kit that has the necessary* ***sensitivity*** *and* ***specificity*** *. Our study confirmed the results of just one previous, recent study using the same BDNF ELISA kit by Ikenouchi and colleagues. This kit is the one BDNF ELISA kit that has been fully validated in a previous study (PMID: 37173369) in which Want and colleagues thoroughly documents both the specificity and sensitivity of this ELISA kit. The other BDNF ELISA kits mentioned by the reviewer report values that are implausibly high for BDNF levels in human saliva, with no demonstration of specificity. We hope that these additional comments will help clarifying what appears to be a misunderstanding about the objective of our study.”*

*In order to clarify our intention, we will modify the Conclusion to:*

 Our results indicate that the BDNF ELISA kit previously validated both with regard to specificity and sensitivity (Want et al.) ^9^ can be used to accurately determine the minute amounts of BDNF present in human saliva. These results also confirm those recently published by Ikenouchi and colleagues ^11^ using the very same BDNF ELISA kit. Correlations can now be readily explored between BDNF levels in human saliva and a range of physiological and pathological conditions of interest, including exercise, ageing, depression and neurodegeneration.

 We would appreciate your kind review.

# Response

**Published:** 2025-04-29

**Authors:** Fumie Akutsu

**Affiliations:** FUJIFILM Wako Pure Chemical Corporation, Japan

Dear Dr. Thomas,

 Please find our responses written below.

**Reviewer’s comments:**

 The paper “A highly sensitive enzyme‑linked immunosorbent assay allows accurate measurements of brain‑derived neurotrophic factor levels in human saliva” presents important assay details for quantifying BDNF levels in saliva samples. These include spike and recovery data for the saliva matrix, the effects of different saliva collection protocols, linearity of dilution in the saliva matrix, precision and reproducibility of the BDNF measures and information regarding sample stability under long-term storage and the effects of multiple freeze thaws on BDNF detection. These are all essential considerations to show that saliva can be used for BDNF measurements. Saliva is a non-invasive biofluid that has gained recent attention for use in a wide range of human studies; hence knowing assay specifics for quantification of a very popular physiological peptide by ELISA has important consequences for others wanting to measure BDNF in saliva.

 The authors list previous studies that have quantified BDNF in human saliva samples and state that “BDNF values reported in these studies varied over a very wide range, indicating that most BDNF measurement methods used thus far had either not the specificity and/or the sensitivity needed to reliably measure BDNF levels in human saliva”.  Aside from using different ELISA assays, it is also very likely that these past studies used different saliva collection methods and storage conditions, which the authors show can affect BDNF levels in saliva. Hence, the current findings could possibly explain some of the variability observed in past studies. This is another relevant aspect of the work.

 A few other corrections/suggestions:

 -In the Introduction, they include Nakagawa et al. (ref 12) and Kikuchi et al., (ref 13) as human saliva studies, when these were carried out in rats. These should be omitted, but there are several other studies that have measured BDNF in saliva samples in humans that could be mentioned.

 à We agree to your comment to remove Nakagawa et al (ref 12) and Kikuchi et al (ref 13), since their studies are not for human but for rat saliva BDNF levels.

 We included the Tahiroglu et al. in the Discussion section (new ref 22) as the most recent report discussing measurement of salivary BDNF.

 -In particular, one past study (Mandel A et al., 2011) also had worked out optimized conditions for another BDNF ELISA (although not as in depth as the current paper) and this study should be mentioned somewhere.

 à We add some description about Mandel A et al in Discussion: “and significant number (up to 35%) of saliva samples needed to be excluded from the analysis due to the lack of appropriate detection sensitivity in the case of Mandel et al ^20^.”

 -In the Abstract, it is not necessary to mention the mouse study: “Recently, the suitability of a highly sensitive BDNF ELISA assay was validated using mouse plasma and serum where conventional BDNF ELISA fail to detect BDNF”. The fact that it was validated in mouse is not as important as the fact that the assay works well in human saliva.

 à Although the reviewer suggests removing the sentence about the mouse study in the Abstract, we believe this is an important improvement in measurement of BDNF in various samples which was not done by commercially available BDNF assays in the past. So, we would like to keep the sentence in the Abstract as it specifically refers to validation, an all-important aspect. Sensitivity is of course key but so is validation. Readers may be interested to go back to the study we cite in our manuscript and check for themselves what is meant by validation. Briefly, beyond the reduction of the BDNF signal using a BDNF monoclonal antibody not used in the BDNF ELISA. the finding that BDNF levels in mouse plasma and serum are indistinguishable is key. Indeed, the process of platelet degranulation leads to the release of a number of cytokines and growth factors from platelets and the finding that the BDNF values remain unchanged compared with the plasma values is an important aspect of the validation.

 -The introductory text regarding the origin of plasma/serum-derived BDNF levels is interesting, as the author make the suggestion that BDNF levels measures in blood might not inform about brain dysfunction in humans. Two previous studies (Ikenouchi A et al., 2023 and Gutierrez A et al., 2020). shown that saliva and plasma levels of BDNF are not correlated, possibly suggesting that saliva measures could be more relevant. This is worth mentioning in the Discussion.

 à Per reviewer’s suggestion, we modify the discussion section: “Obviously, saliva samples can be readily and repeatedly collected for BDNF level determinations and possible correlations explored with various neurological and psychiatric conditions. While the origin of BDNF in saliva is unclear, the study by Ikenouchi et al. ^12^ demonstrated that there is no correlation between the levels of mBDNF in saliva and plasma. One possible source of BDNF in saliva samples are the sensory nerves innervating the buccal cavity including the tongue and gingiva as the levels of BDNF in sensory afferents are comparatively high (for review, see 2). Given that it is now possible to measure BDNF levels in human saliva, it would be interesting to know whether BDNF levels in saliva correlate with a range of physiological and pathological conditions of interest using appropriate study designs.”  

 -The time frame (i.e. 2 pm to 5 pm) of saliva sample collection should be stated.  Also, the amount of saliva (i.e. 1 ml) collected should be stated.

 Is the work clearly and accurately presented and does it cite the current literature?

 à We modify the part about the sample collection by adding the time frame and amount of the saliva sample collection to the original manuscript: All samples were collected in the same afternoon (noon to 5 pm) and then 6 to 10 mL of the samples were aliquoted into 1.5 mL
